# Impact of Cold Radiofrequency Air Plasma Treatment on the Bulk Properties of Polypropylene Films

**DOI:** 10.3390/ma19040693

**Published:** 2026-02-11

**Authors:** Artem Gilevich, Oleg Gendelman, Yuri Mikhlin, Shraga Shoval, Edward Bormashenko

**Affiliations:** 1 Chemical Engineering Department, Faculty of Engineering, Ariel University, P.O. Box 3, Ariel 407000, Israel; artgilevich@gmail.com; 2 Faculty of Mechanical Engineering, Technion, Haifa 3200003, Israel; ovgend@me.technion.ac.il; 3 Department of Chemistry, Bar-Ilan University, Ramat Gan 5290002, Israel; yury.mikhlin@biu.ac.il; 4 Department of Industrial Engineering and Management, Faculty of Engineering, Ariel University, P.O. Box 3, Ariel 407000, Israel; shraga@ariel.ac.il

**Keywords:** polypropylene, plasma treatment, bulk properties, toughness, surface properties, relaxation, inverse Rehbinder effect

## Abstract

Extruded polypropylene (PP) films were exposed to cold air plasma treatment, which resulted in significant changes in their bulk properties. The maximal elongation, ultimate tensile strength (UTS), and toughness of the films were increased. The toughness of the films increased from $U_{T0}=(3323\pm 400)\;MPa$ to $U_{T\_PT}=(4434\pm 400)\;MPa$, which is due to the growth of both the maximal elongation and the UTS of the plasma-treated samples. We relate the improvement of the mechanical properties of PP to the morphological transformations revealed in the plasma-treated PP films. Plasma treatment of PP samples was also followed by the modification of their surface properties. Plasma treatment resulted in hydrophilization of PP films followed by hydrophobic recovery. The bulk and surface properties of the plasma-treated PP films evolve with time. The following hierarchy of the temporal scales related to the studied relaxation processes is established: $\tau_{HR}>\tau_{\varepsilon }=\tau_{T}=\tau_{UTS}>\tau_{E}$, where $\tau_{HR},\;\tau_{\varepsilon },\;\tau_{T},\;\tau_{UTS}$ and $\tau_{E}$ are the time scales of the change in the apparent contact angle (hydrophobic recovery), elongation, toughness, ultimate tensile strength, and Young modulus, respectively. The longest of the relaxation times is related to the surface processes, i.e., hydrophobic recovery. The stress–strain curves of the untreated virgin and plasma-treated PP are well described with the twin-slope linear dependencies. The post-plasma-treatment recovery of the tangent modulus is reported. Cold plasma treatment of polypropylene produces surface oxidation and functionalization, evidenced by the emergence of C–O, C=O, and COOH functionalities.

## 1. Introduction

Polypropylene (PP) is among the most widely used thermoplastic polymers due to its low cost, low density, good mechanical properties, biocompatibility, and excellent chemical resistance [1,2,3,4,5]. These characteristics have made PP indispensable in packaging, textiles, automotive components, and biomedical devices. However, like many polyolefins, PP is chemically inert and possesses a low surface energy, typically in the range of 29–31 $\frac{mJ}{m^{2}}$ [6]. This inherent hydrophobicity results in poor wettability and weak adhesion to coatings, inks, dyes, or adhesives, limiting its applicability in processes where strong interfacial bonding is required [7,8,9].

Surface modification is a widely adopted strategy to overcome these limitations without compromising the desirable bulk properties of the PP [10,11,12,13,14,15,16,17,18]. Among the available techniques, such as flame treatment [15], corona discharge [15], UV, ozone irradiation [16], and chemical etching [17], cold plasma treatment has attracted increasing attention due to its environmental compatibility, tunability, and ability to modify only the outermost molecular layers of the material [10,11,12,13]. Unlike thermal plasmas, cold plasmas operate at near-ambient temperatures, which allows treatment of thermally sensitive polymers without essential modification of their bulk structure.

Cold plasma treatment introduces a variety of reactive species, including ions, electrons, free radicals, and UV photons, which interact with the polymer surface through a combination of physical and chemical processes [19,20,21,22,23,24,25,26,27,28,29,30]. These interactions may lead to surface cleaning, crosslinking, chain scission, and, most importantly, to the incorporation of polar functional groups such as hydroxyl, carbonyl, or carboxyl moieties [30,31,32,33,34,35,36,37,38,39,40]. As a result, the surface energy of PP can be significantly increased, enhancing its wettability, printability, and adhesion properties [41,42]. The degree and nature of surface modification depend strongly on the plasma parameters such as gas composition, power, pressure, treatment time, and electrode configuration [41,42].

In recent decades, numerous studies have demonstrated that cold plasma treatment is an effective, solvent-free, and rapid method for tailoring PP surfaces for specific applications, ranging from improved adhesion in composites to enhanced biocompatibility for medical devices [9,10,11,12,13,14,15,16,26,36,41,42]. Nevertheless, the precise relationship between plasma conditions, surface chemistry, and long-term stability of the induced modifications remains an active area of research. In particular, the phenomenon of hydrophobic recovery—partial reversion of treated PP surfaces toward their original low-energy state over time—poses challenges for practical implementation [43,44,45,46,47,48,49,50,51,52,53].

The present work investigates the effects of cold plasma treatment on PP films under controlled conditions, with particular emphasis on plasma-induced changes in bulk material properties. To date, only a limited number of studies have explored the modification of polymer bulk properties using cold plasma [52,53]. Although these studies report promising results, the underlying plasma–polymer interactions and the resulting changes in bulk properties have not been systematically investigated. A clear understanding of the relationship between plasma processing parameters and the resulting surface and bulk characteristics is essential for designing effective and durable plasma-based modification strategies for polypropylene in industrial applications. Here, we demonstrate that cold plasma treatment produces significant modifications not only in the surface but also in the bulk properties of PP films.

## 2. Materials and Methods

Extruded lab-made polypropylene (PP) film samples with dimensions of 150 × 20 mm and thickness 30±1 μm were cut using a scalpel blade, and then washed sequentially with acetone, water, ethanol, and finally water. The washed samples were dried for 24 h at ambient conditions. Analytical acetone and ethanol were supplied by Sigma-Aldrich Israel. The de-ionized water was purified by a synergy UV water purification system from Millipore SAS (Molsheim, France) and its specific resistivity was $\hat{\rho }=18.5\;M\Omega \times cm$ at 25 °C

Plasma treatment was carried out in the closed radiofrequency (RF) plasma installation PDC-32G manufactured by MTI Co., Pleasantville, NY, USA. The power of the air plasma was 11 W, the plasma frequency was 13.56 MHz, the pressure in the plasma chamber was on average 1 Torr (133 Pa) (as established with BOC Edwards Barocel Pressure Sensor 655AB 10TR, USA), and the plasma electron temperature was approximately 10^4^ K.

The electron density of plasma corresponding to 1 Torr is approximately $10^{9}\;{cm}^{-3}$ (data supplied by Harrick Scieintific Co.; see also: [49]). Time of exposure was fixed at 30 s. Longer time spans of plasma treatment resulted in the deformation of the sample. After the plasma treatment, the samples were exposed to the environment: the average temperature was 22–23 °C.

Air was supplied by Yakov Salam and Banav Ltd. (Israel). The air contained 78% $N_{2}$, 21% $O_{2}$, and 1% impurities $({CO}_{2},\;Ar$, and $H_{2}O$).

A series of experiments was carried out in order to identify the effect of cold plasma on the mechanical and wetting properties of PP and their relaxation. The first set of samples was tested without plasma exposure. The remaining samples after plasma treatment were tested for 48 h at intervals.

**Figure 1:**
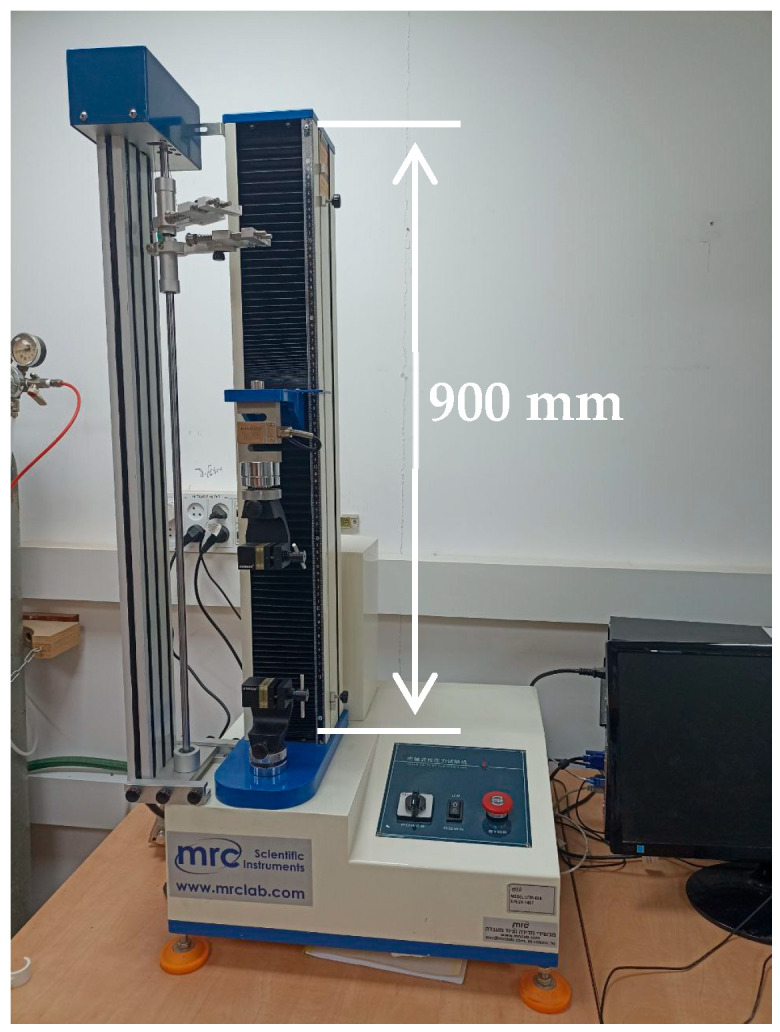
The laboratory Tensile Testing Machine UTM-65A used in the investigation. Test stroke: 650 mm (not including grips); resolution: 1/250,000; force accuracy: ∆f < ±0.5%.

The mechanical measurements were performed on a Tensile Testing Machine UTM-65A, manufactured by MRC Lab (MRC Ltd., Holon Israel), supplied by M.R.C. (Rachmanov & Bookstein, Ltd., Israel), with a maximum load of 100 N (Figure 1).

The tensile test speeds were 50 mm/min and 10 mm/min. For each time span of plasma treatment, 13–16 measurements were taken. After that, the basic mechanical properties were established, including Young modulus (*E*), ultimate tensile strength (UTS), toughness, and yield stress.

Plasma effect on the surface energy was verified by measuring the apparent water contact angle. The experiment was performed on a Ramé-Hart Advanced Goniometer Model 500-F1 (Ramé-Hart, USA). For each sample, approximately 18 measurements were performed along the length of the sample. The water droplet volume was 5 μL.

The FTIR spectrum of the PP was measured in the transmission mode (bulk test) using a Mid-IR FTIR spectrometer JASCO FT/IR-4600 (JASCO, Tokyo Japan) at ambient conditions, with a resolution of $1.0\;{cm}^{-1}$, and is supplied in Appendix A.

**Figure A2:**
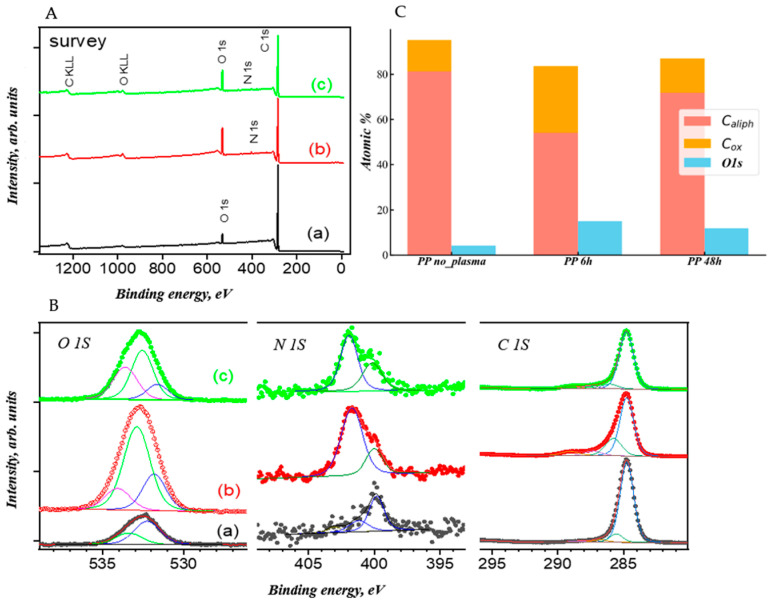
Inset (**A**): (a) XPS spectrum for virgin PP films (i.e., not treated with plasma, denoted “no_plasma”) is depicted. Spectra (b,c) are established for PP films after 6 and 48 h after plasma treatment, respectively. Inset (**B**): Gaussian peak decomposition of elemental peaks. XPS spectrum reveals temporal evolution of elemental composition in plasma-treated PP. Inset (**C**): Evolution of the XPS spectrum. Increase in oxygen content and corresponding decrease in carbon content following plasma treatment is recognized.

X-ray photoelectron spectra (XPS) were conducted using a ThermoScientific Nexsa G2 instrument (Waltham, MA, USA) survey, and high-resolution spectra were obtained at pass energies of 200 and 50 eV respectively, using a monochromatic Al Kα X-ray source (*hν* = 1486.6 eV, spot size 400 μm) and a hemispherical electron energy analyzer (128 channels). To mitigate sample charging, an electron flood gun was used. All binding energies were referenced to the C 1s peak of aliphatic carbon at 284.8 eV. The resulting data (Figure A2) were processed with Avantage 6.9 software (ThermoScientific) and are presented in Appendix B.

Three kinds of PP films were analyzed with XPS, namely: untreated virgin PP films, 6 h post-plasma treatment, and 48 h post-treatment PP films. Measurements in different points showed no significant compositional variations, indicating relatively homogeneous surface modification.

## 3. Results

### 3.1. Stress–Strain Curves Registered for the Virgin and Plasma-Treated PP Films

**Figure 2:**
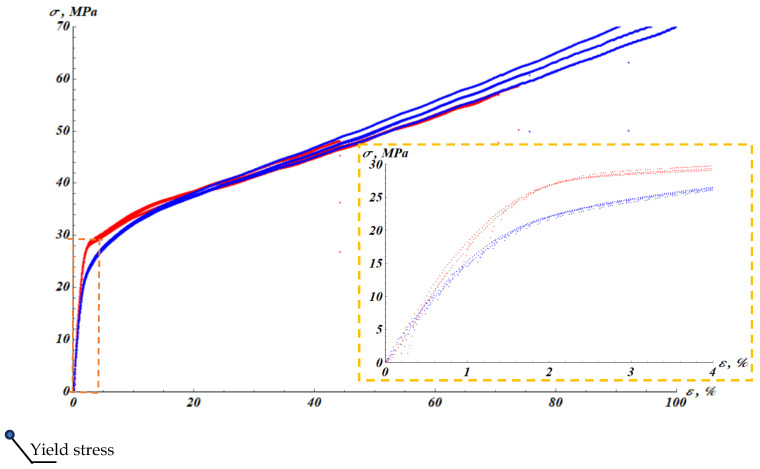
Stress (*σ*)–strain (*ε*) experimental dependencies for non-plasma-treated samples (red curves) and for samples tested right after plasma treatment (blue curves). The inset (dash box) depicts the initial stage of the deformation.

The raw results of mechanical tests are shown in Figure 2, depicting the stress–strain dependencies $\sigma \left(\varepsilon \right)$ for virgin and plasma-treated PP samples.

**Figure 3:**
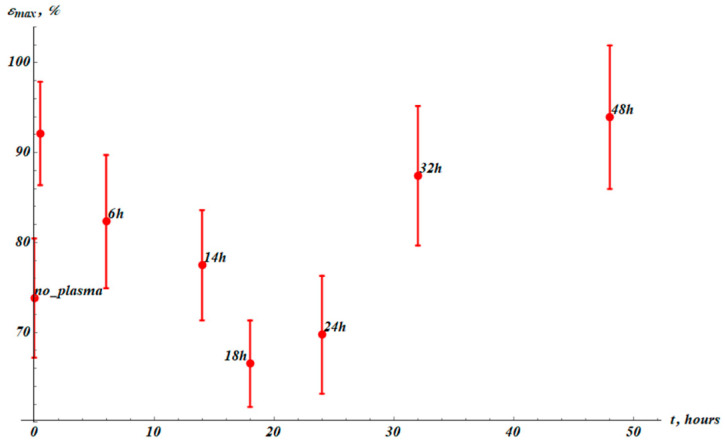
Maximum elongation ($\varepsilon_{max}$) values established from stress–strain experimental curves. The point labeled “no_plasma” corresponds to the virgin samples not treated with plasma. The next point (without label) corresponds to the $\varepsilon_{max}$ measured right after plasma treatment. The results were averaged over 15 samples.

Now examine the impact of the cold plasma treatment on the maximal elongation of the samples $\varepsilon_{max}$, as depicted in Figure 3.

The maximal elongation $\varepsilon_{max}$ is markedly increased from $\varepsilon_{max0}=(74\pm 7)\%$ for the untreated samples to $\varepsilon_{maxPT}=(92\pm 6)\%$ for the plasma-treated samples. The temporal dependence $\varepsilon_{max}\left(t\right)$ demonstrates the following behavior: it increases immediately after the plasma treatment of the sample, drops to a minimum, and then recovers to the initial post-treatment value after $\tau_{\varepsilon r}≅50\;h$ of the recovery. The characteristic time scale of the elongation change is estimated as $\tau_{\varepsilon }=\frac{1}{2}\tau_{\varepsilon r}≅25\;h.$

**Figure 4:**
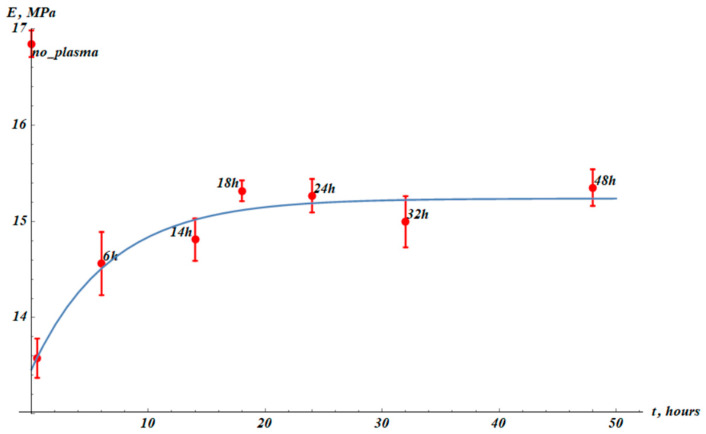
Young modulus (*E*) values (red points) established from experimental stress–strain curves (see Figure 2). The point denoted “no_plasma” corresponds to the samples not treated with plasma. The next point (without label) corresponds to measurements immediately after plasma treatment. The blue fitting curve corresponds to Equation (1) with fitting parameters: $E_{\infty }=(15.2\pm 0.1)\;MPa,\;$∆E=(1.8±0.2) MPa, $\tau_{E}=(6.7\pm 2.4)\;h$. Squared root-mean-square correlation coefficient (squared Pearson correlation coefficient) for the exponential fitting of the experimental data with Equation (1) is $R^{2}=0.99982.$ The results were averaged over 15 samples.

The Young modulus of the samples also changed markedly, with the post-treatment dwell time denoted *t* (time measured after the plasma treatment of the PP sample). The dependence of the Young modulus on the post-treatment dwell time, $E\left(t\right)$, is depicted in Figure 4. The initial Young modulus $E_{0}$ dropped from $E_{0}=(16.8\pm 0.1)\;MPa$ to $E_{PT}=(13.6\pm 0.2)\;MPa$ as measured immediately after the plasma treatment.

We used the following approximation formula for Young modulus (Equation (1)):

(1) $$E\left(t\right)=E_{\infty }-∆E\cdot exp\left(-\frac{t}{\tau_{E}}\right),$$

where E (Young modulus) and *t* were taken from experiment data. The fitting parameters ($E_{\infty },\;∆E,\;and\;\tau_{E}$) were found using nonlinear regression. The best-fit parameters are $E_{\infty }=(15.2\pm 0.1)\;MPa,$ ∆E=(1.8±0.2) MPa, $\tau_{E}=(6.7\pm 2.4)\;h$.

We conclude that exponential fitting satisfactorily describes the time evolution of the Young modulus of the cold plasma treatment of PP samples. The time span $\tau_{E}$ is reasonably understood as the characteristic time of the temporal change in the Young modulus of the samples. Comparing the experimental data presented in Figure 3 and Figure 4, we conclude that the interrelation $\tau_{\varepsilon }\gg \tau_{E}$ takes place. In other words, the characteristic time span of the change in the maximal elongation of the sample is much larger than that of the Young modulus. This observation hints at the hierarchy of the relaxation processes occurring in the cold-air-plasma-treated PP films.

**Figure 5:**
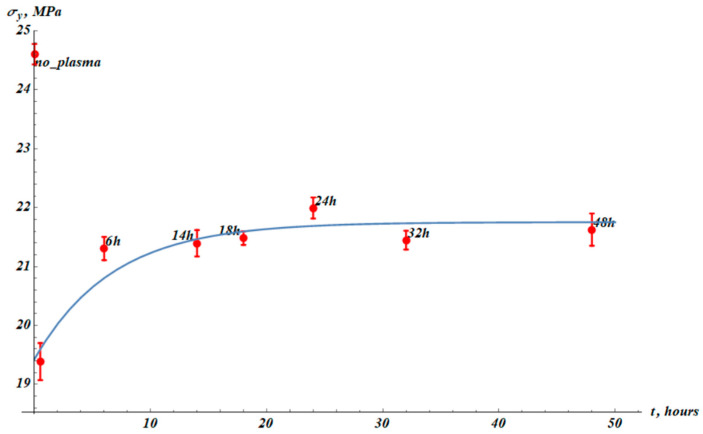
Yield stress ($\sigma_{y}$) values (red points) established from experimental stress–strain curves (see Figure 2). The point “no_plasma” corresponds to the samples not treated with plasma. The next point (without label) corresponds to measurements immediately after plasma treatment. The blue fitting curve corresponds to Equation (2) with fitting parameters: $\sigma_{y\infty }=\left(21.8\pm 0.2\right)\;MPa$, $∆\sigma_{y}=(2.32\pm 0.4)\;MPa$. The squared Pearson correlation coefficient $R^{2}=$ 0.999. The results were averaged over 15 samples.

It can be noticed that yield stress temporal evolution behaves similarly to the Young modulus. The characteristic time of the change in the yield stress, $\tau_{YS}$, is close to that of the Young modulus recovery (see Appendix C); $\tau_{YS}\approx \tau_{E}$. Therefore, we assume the same functional form for the fitting formula (Equation (2)) with the same value $\tau_{E}=6.7\;h$:

(2) $$\sigma_{y}(t)=\sigma_{y\infty }-∆\sigma_{y}\cdot exp\left(-\frac{t}{\tau_{E}}\right),$$

where $\sigma_{y\infty }$ and $∆\sigma_{y}$ are fitting parameters, and $\sigma_{y}$(*t*) represent experimental yield stress data (Figure 5). The best-fit parameters are $\sigma_{y\infty }=(21.8\pm 0.2)\;MPa,$ $∆\sigma_{y}=(2.3\pm 0.4)\;MPa.$ The squared Pearson correlation coefficient is $R^{2}=0.999$. It is noteworthy that in our case the yield stress is very close to the proportional limit. The yield stress of the virgin samples is $\sigma_{y\_0}=\left(24.6\pm 0.2\right)\;MPa;$ the yield stress immediately after the plasma treatment is $\sigma_{y\_PT}=(19.4\pm 0.3)\;MPa$.

We recognize that $\sigma_{y\infty }<\sigma_{y\_0}$. Thus, the temporal recovery of the yield stress is not complete. Now we examine the stress–strain curve $\sigma \left(\varepsilon \right)$ depicted in Figure 2. The curve depicted in Figure 2 is well-represented by a bilinear (two-slope) mechanical model. The elastic region (first slope) is described by Equation (3):

(3) $$\sigma =E\varepsilon ,\;\varepsilon \leq \varepsilon_{y},$$

where $\varepsilon_{y}$ is the yield strain (see Appendix C) corresponding to the yield stress $\sigma_{y}$. It is recognized from the data supplied in Appendix C that $\varepsilon_{y}$ is practically independent of plasma treatment.

Beyond yield, the stress–strain curve has another slope, smaller than *E*. This is the tangent modulus or strain-hardening modulus, usually denoted $E_{t}$. The plastic deformation is described by Equation (4):

(4) $$\sigma =\sigma_{y}+E_{t}\left(\varepsilon -\varepsilon_{y}\right),\;\varepsilon >\varepsilon_{y}$$

**Figure 6:**
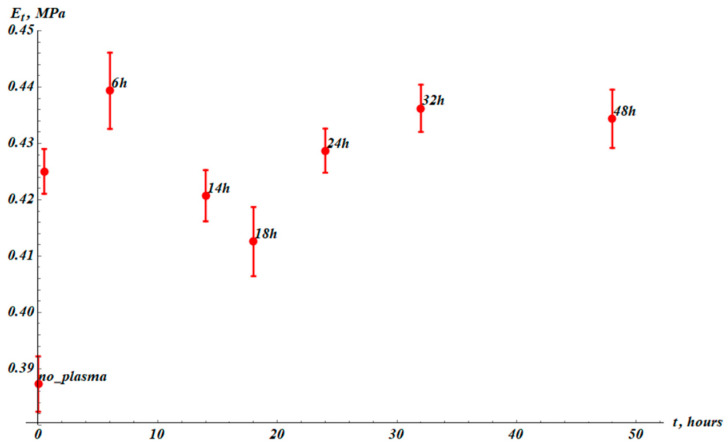
Tangent modulus ($E_{t}$) values established from stress–strain experimental curves. The point “no_plasma” corresponds to the samples not treated with plasma. The next point (without label) corresponds to measurements immediately after plasma treatment. The results were averaged over 15 samples.

**Figure 7:**
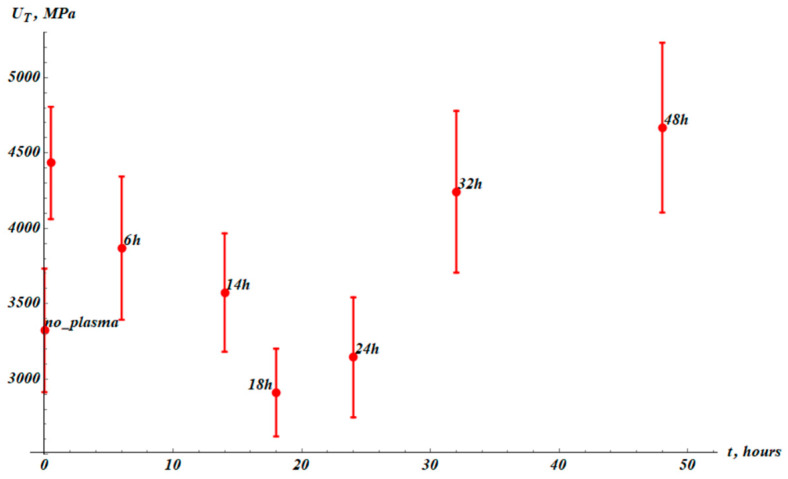
Toughness ($U_{T}$) established as integral function (area under the curve) of stress–strain experimental curves. The point “no_plasma” corresponds to the virgin samples not treated with plasma. The next point (without label) corresponds to measurements immediately after plasma treatment. The results were averaged over 15 samples.

Let us discuss the time dependence of the tangent modulus labeled $E_{t}$. The tangent modulus is increased by plasma treatment from $E_{t\_0}=(0.387\pm 0.005)\;MPa$ to $E_{t\_PT}=(0.425\pm 0.004)\;MPa,$ as shown in Figure 6. Afterwards, the temporal behavior of $E_{t}$ follows the same pattern as $\varepsilon_{max}$, with the same characteristic time of total recovery $\tau_{\varepsilon r}\approx 50\;h$ (compare Figure 3 and Figure 6). The characteristic time scale of the tangent modulus change was established as $\tau_{tm}\approx 20\;h$, close to that of maximal elongation. This result is quite reasonable: the recovery of the plastic properties of the samples is governed by the same mechanism. We now examine the temporal dependence of toughness, denoted $U_{T}$ and illustrated in Figure 7.

### 3.2. Toughness of the Virgin and Plasma-Treated PP Films

Recall that toughness, denoted $U_{T}$ is defined by Equation (5):

(5) $$U_{T}=\int_{0}^{\varepsilon_{max}}\sigma \left(\varepsilon \right)d\varepsilon$$

Toughness $U_{T}$, given by Equation (5), is the energy absorbed per unit volume by a specimen up to fracture. Numerically, it is the area under the stress–strain curve from zero strain to the fracture strain. The toughness is increased as a result of the plasma treatment, from the $U_{T0}=\left(3323\pm 400\right)\;MPa$ to $U_{T\_PT}=(4434\pm 400)\;MPa$. This is a very important result revealed in our investigation. The characteristic time scale of the toughness change was established as $\tau_{T}\approx 25\;h$, and is very close to that of maximal elongation $\tau_{\varepsilon }$.

**Figure 8:**
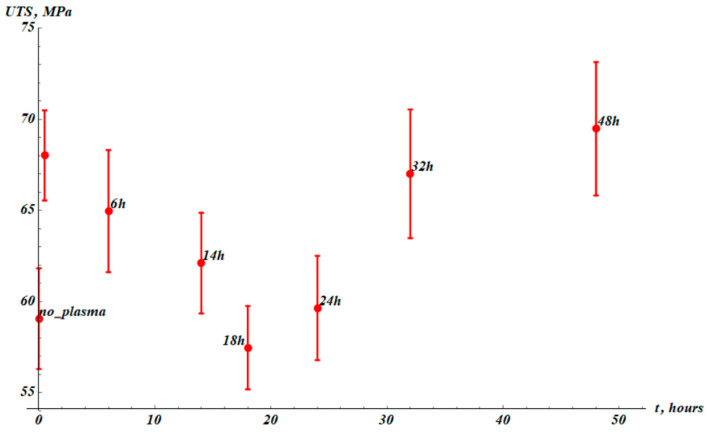
The UTS (UTS) values established from stress–strain experimental curves. The point “no_plasma” corresponds to the virgin samples non-treated with plasma. The next point (without label) corresponds to measurements immediately after plasma treatment. The results were averaged over 15 samples.

Now address the ultimate tensile strength of the samples, abbreviated as UTS and depicted in Figure 8.

The UTS increased as a result of cold plasma treatment from ${UTS}_{0}=(59\pm 3)\;MPa$ to ${UTS}_{PT}=(68\pm 2)\;MPa$. The temporal behavior of the tangent modulus ($E_{t}$), toughness ($U_{T}$), and UTS was the same (see Figure 6, Figure 7 and Figure 8); $\tau_{UTS}\approx \tau_{T}\approx \tau_{\varepsilon }\approx 25\;h$.

It should be emphasized that the aforementioned time dependencies of the mechanical characteristics of plasma-treated PP were the same for tensile speeds $v_{T}$ of 10 or 50 mm/min.

### 3.3. Surface Properties of the Virgin and Plasma-Treated PP Films

**Figure 9:**
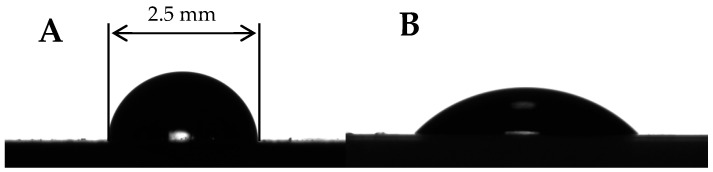
Water droplet (5 μL) placed on the PP samples: (**A**) untreated PP sample, (**B**) after plasma treatment. Scale bar is 2.5 mm.

Now we observe the time dependence of the apparent water contact angle established for the plasma-treated PP. Plasma treatment decreased the apparent contact angle of PP, as depicted in Figure 9. The initial apparent contact angle was established as $\theta_{0}=103.0{}^{\circ}\pm 0.3{}^{\circ}$. The apparent contact angle after the plasma treatment was $\theta_{PT}=53.5{}^{\circ}\pm 2.2{}^{\circ}$. These results are close to those reported for PP films treated by diffuse coplanar surface barrier discharge [50].

**Figure 10:**
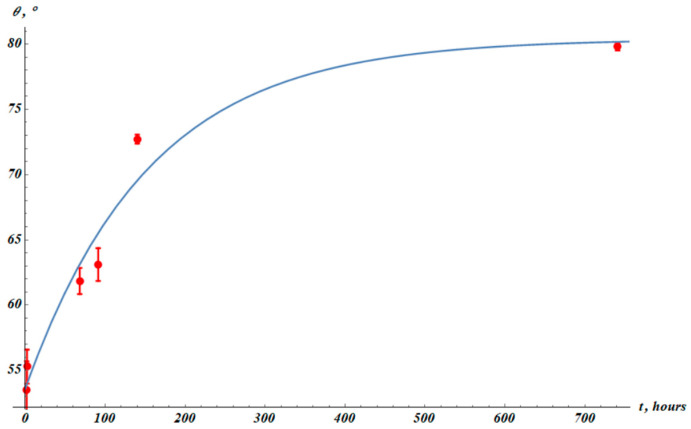
Apparent contact angle (θ) experimental data (red points). The blue fitting curve corresponds to Equation (6) with fitting parameters: $\theta_{\infty }=80.4{}^{\circ}\pm 2.7{}^{\circ}$, ∆θ=26.8°±3.2°, $\tau_{HR}=(155\pm 41)\;h$. Squared Pearson correlation coefficient $R^{2}=0.998$.

The apparent contact angle was restored with time (this process is known as hydrophobic recovery) [43,44,45,46,47]. Hydrophobic recovery is illustrated in Figure 10. The phenomenological fitting formula describing hydrophobic recovery (Equation (6)) is exponential:

(6) $$\theta (t)=\theta_{\infty }-∆\theta \cdot exp\left(-\frac{t}{\tau_{HR}}\right),$$

where $\theta_{\infty }$, ∆θ, and $\tau_{HR}$ are fitting parameters, and $\theta \left(t\right)$ represents the measured apparent angle (Figure 10). The best-fit parameters are $\theta_{\infty }=80.4{}^{\circ}\pm 2.7{}^{\circ},$ ∆θ=26.8°±3.2°, $\tau_{HR}=(155\pm 41)\;h.$ Squared Pearson correlation coefficient: $R^{2}=0.998$.

**Figure 11:**
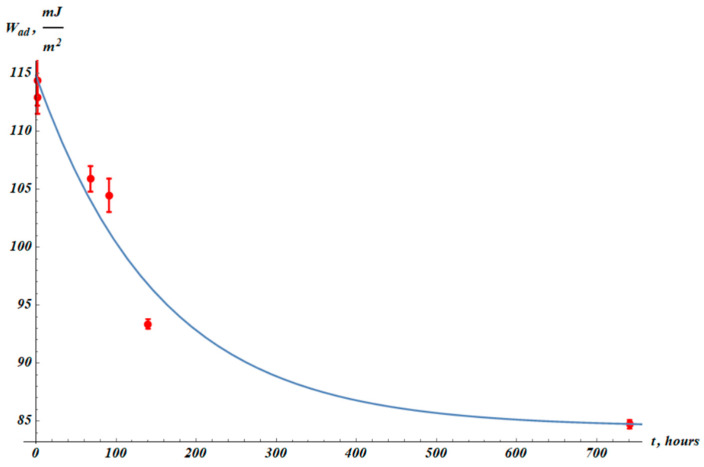
The work of adhesion ($W_{ad}$) values established from apparent contact angle measurements (Figure 10). The blue fitting curve corresponds to Equation (8) with fitting parameters: $W_{ad\_\infty }=(84\pm 2)\;\frac{mJ}{m^{2}}$, $∆W_{ad}=(30\pm 3)\;\frac{mJ}{m^{2}}$, $\tau_{HR}=155\;h.$ Squared Pearson correlation coefficient: $R^{2}=0.999$.

It was also instructive to establish the temporal dependence of the work of adhesion. The work of adhesion is defined as the work necessary for separation of a water droplet from a given surface [50]. The work of adhesion, denoted $W_{ad}$, is calculated according to the Young–Dupre equation:

(7) $$W_{ad}=\gamma \left(1+\cos\theta \right),$$

where $\gamma =71\;\frac{mJ}{m^{2}}$ is water surface tension, and θ is the Young equilibrium contact angle [51]. The Young equilibrium contact angle established on the extruded polymer films is very close to the goniometrically established apparent contact angle, actually taken for the calculation of $W_{ad}$. The work of adhesion for the virgin PP samples is $W_{ad\_0}=(55.8\pm 0.4)\;\frac{mJ}{m^{2}}$. The adhesion work of plasma-treated films is $W_{ad\_PT}=(114\pm 2)\;\frac{mJ}{m^{2}}$. The fitting formula describing hydrophobic recovery is exponential:

(8) $$W_{ad}\left(t\right)=W_{ad\_\infty }+∆W_{ad}\cdot exp\left(-\frac{t}{\tau_{HR}}\right),$$

where $W_{ad\_\infty }$, $∆W_{ad}$ and $\tau_{HR}$ are fitting parameters (see Figure 11). Relaxation time $\tau_{HR}=155\;h$ is the same as for apparent contact angle. The best-fit parameters are $W_{ad\_\infty }=(84\pm 2)\;\frac{mJ}{m^{2}},$ $∆W_{ad}=(30\pm 3)\;\frac{mJ}{m^{2}}.$ Squared Pearson correlation coefficient: $R^{2}=0.999$.

Increase in the work of adhesion is at least partially attributed to the change in the roughness of the plasma treated PP films [50].

## 4. Discussion

### 4.1. Main Experimental Findings and Their Interpretation

The main experimental findings of this study can be summarized as follows.

(i) Cold air plasma treatment of PP films leads to substantial changes in their bulk properties. The maximal elongation, UTS, and toughness of the films are essentially increased by the plasma treatment. This is the most surprising and important result of our investigation, demonstrating potential for industrial applications of plasma-treated PP. The toughness of the films was markedly increased as a result of the plasma treatment, from $U_{T0}=(3323\pm 400)\;MPa$ to $U_{T\_PT}=(4434\pm 400)\;MPa$. This increase is due to the growth of both the maximal elongation and UTS of the plasma-treated samples. We do not explore in this paper the microscopic processes occurring in the plasma-treated PP films; however, it is reasonable to relate the improvement of mechanical properties of PP to the morphological transformations revealed in plasma-treated PP [52]. The virgin isotactic polypropylene is composed in bulk of smectic (partially ordered, liquid crystalline-like) and amorphous phases in the same proportions, and after the plasma treatment, a more organized monoclinic α—phase was registered [52]. It was also reported that as a result of the nitrogen plasma treatment, two types of crystallization occurred, namely, the smectic phase transformed into the α—phase, and the amorphous phase transformed into the smectic phase [52]. This mechanism reasonably explains the changes in the bulk mechanical properties of the plasma-treated PP. Regrettably, the authors of Ref. [52] did not report the mechanical testing of plasma-treated PP, which is provided in our paper. An increase of crystallinity index of polymers was reported following the cold plasma treatment, as evidenced with the XRD study [53].

Dramatic changes in bulk properties of solids due to their surface treatment are known in materials science as the Rehbinder effect [54,55,56,57]. The Rehbinder effect (or Rehbinder phenomenon) refers to a reduction in the mechanical strength (or hardness) of a solid material due to adsorption of surface-active substances (surfactants, liquids, or gases) on its surface [54,55,56,57]. The adsorbed molecules modify the surface energy of a solid [54,55,56,57]. This influences dislocation motion and crack propagation—the key processes that control plastic deformation and fracture. In contrast, we observed the “inverse Rehbinder effect”, whereby the surface plasma treatment improves the bulk mechanical properties of PP. The physical mechanism of the “direct” and “inverse” Rehbinder effects are different, since the mechanical properties of crystals and polymers are governed by very different physical laws [58].

(ii) Plasma treatment of PP samples is followed by the temporal evolution of their mechanical and surface properties. The following hierarchy of the temporal scales is established: $\tau_{HR}>\tau_{\varepsilon }=\tau_{T}=\tau_{UTS}>\tau_{E}$, where $\tau_{HR},\;\tau_{\varepsilon },\;\tau_{T},\;\tau_{UTS}$, and $\tau_{E}$ are the time scales of the change in apparent contact angle, elongation, toughness, ultimate tensile strength, and Young modulus respectively (see Table 1). This means that a diversity of processes is involved in the relaxation of the plasma-treated PP films. The slowest among them are the surface processes resulting in the time evolution of the apparent contact angle and adhesion energy.

**Figure A3:**
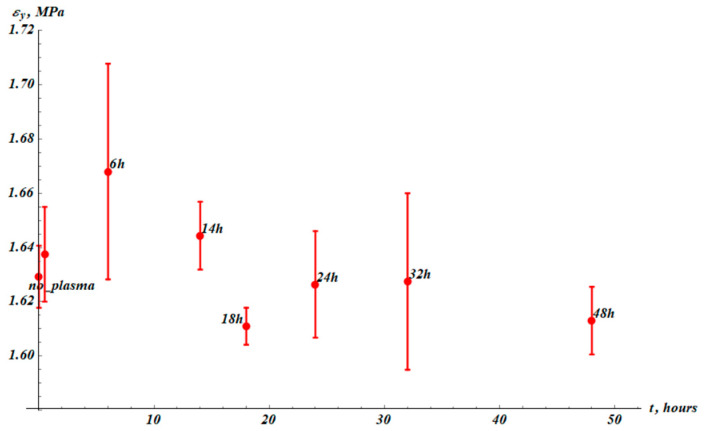
Yield strain ($\varepsilon_{y}$) values (red points) established from experimental stress–strain curves (see Figure 2). The point “no_plasma” corresponds to the samples not treated with plasma. The next point (without label) corresponds to measurements immediately after plasma treatment.

Generally speaking, the relaxation of polymers may be described by Equation (9):

(9) $$f\left(t\right)=\sum_{i=1}^{n}A_{i}exp\left(-\frac{t}{\tau_{i}}\right)$$

where $f\left(t\right)$ is the time-dependent physical parameter (Young modulus, toughness, etc.) and $A_{i}$ are the constant coefficients representing the contribution of the *i*-th relaxation process with the corresponding relaxation time $\tau_{i}$ [59,60,61]. Recovery of the relaxation spectrum from given experimental data is a non-trivial and challenging mathematical problem [62,63,64,65,66,67]. In a number of experimental situations, the relaxation spectrum of the polymer may be reasonably represented by the triad of relaxation times [67]. We restrict ourselves to this simplified model, considering the triad of relaxation times, namely: $\tau_{HR},\;\tau_{T}$, and $\tau_{E}$. It is noteworthy that the longest of the relaxation times is related to the surface processes (i.e., hydrophobic recovery). We do not assert that the triad of relaxation times $\tau_{HR},\;\tau_{T}$, and $\tau_{E}$ completely describes the relaxation spectrum of the plasma-treated PP; these are the relaxation time spans extracted from our experimental data.

(iii) The stress–strain curves of the virgin and plasma-treated PP are well described by the twin-slope linear dependencies. The tangent modulus $E_{t}$ of the plasma-treated PP is increased by plasma treatment. The tangent modulus of the plasma-treated samples evolves with time. The characteristic time scale of the tangent modulus change was established as $\tau_{tm}\approx 20\;h$, close to that of the maximal elongation.
(iv) There are no changes in the FTIR transmission spectra of plasma-treated PP films when compared to virgin ones (see Appendix A). More sensitive experimental techniques should be involved for revealing changes in the morphology and chemical composition of plasma-treated PP.
(v) Plasma treatment induced substantial modifications in surface elemental composition (Figure A3). The untreated PP exhibited 95.1 at% carbon and 4.2 at% oxygen (originating from adventitious carbonaceous contamination and minor oxidation of polypropylene surface). For 6 h post-treatment, carbon content decreased to an average of 85 at%, while oxygen increased dramatically, to 11–12 at% (Figure A3). In addition, XPS found about 1 at% nitrogen and negligible, in the order of 0.2 at%, contaminations of phosphorus (phosphate-like groups with P 2p peak at 133.8 eV), and sulfur. The surface composition insignificantly, e.g., oxygen within ±2 at%., varied in various points, and remained almost the same after several hours in the ultrahigh vacuum. After air aging for 48 h, a minor recovery of the plasma-treated PP was observed, with carbon content slightly rising to 87.2 at% and oxygen stabilizing at 11.5 at%, and nitrogen at about 1–1.2 at%.

High-resolution C 1s spectra revealed the formation of oxygen-containing functional groups following the plasma treatment. The untreated sample showed predominantly aliphatic carbon (284.8 eV) with minor oxidized components. The C 1s spectra of post-treatment samples can be fitted with maxima attributable to C–O and C–N bonds (~286 eV, about 18 at% at point 1), carbonyl groups C=O (287.3 eV, about 5%), and carboxyl moieties O–C=O (289.1 eV, 5 at%). The O 1s band enhanced after the treatment mainly due to the contribution of COH groups (BE of 532.6 eV) and adsorbed water (533.6 eV); however, fitting the C 1s and O 1s spectra is not fully unequivocal. The N 1s spectrum of the initial film showed a weak line of amine/amide groups (~400 eV), and a peak emerging after the plasma treatment at ~402 eV, possibly from imide groups [-], consistent with nitrogen incorporation from air plasma. The components persisted at 48 h air-aged samples, confirming stable surface functionalization.

(vi) Surface oxidation of plasma treated PP is practically irreversible, whereas the established apparent contact angle is practically reversible with time. Thus, the initial dramatic change in the apparent contact angle is not totally due to the surface oxidation.
(vii) The time scales of hydrophobic recovery and changes in the bulk properties of plasma treated PP are well-separated. Thus, it is reasonable to conclude that the bulk and surface processes occurring in the plasma-treated PP films are driven by different physical mechanisms. The mechanism of hydrophobic recovery remains mysterious to a great extent. It is mainly related to (1) reorientation of polar groups formed by the plasma treatment on the surface of the polymer, (2) diffusion of low-molecular-weight oxidized entities, and (3) radical recombination. The long relaxation times for the relaxation of the contact angle is evidence for the diffusion mechanism of the hydrophobic recovery [45].

We summarize the novelty of the manuscript as follows: we demonstrate that cold RF air plasma, traditionally considered a surface-only treatment, induces a strong, time-dependent enhancement of polypropylene bulk mechanical properties (inverse Rehbinder effect) with a clear hierarchy of relaxation time scales separating bulk recovery from hydrophobic recovery.

### 4.2. Trends for Future Investigations

In our future investigations we plan:

(i) To investigate the changes in the chemical structure of the plasma-treated PP films.
(ii) To study XRD spectra of the plasma-treated PP films.
(iii) To investigate the morphological changes in the plasma-treated PP films.
(iv) To study the cyclical plasma treatment of the PP films.
(v) To study the possibilities of controlling the recovery of surface and bulk properties of PP films. In particular, immersion of the plasma-treated films in polar liquids may influence this recovery.
(vi) To study the impact of different gaseous plasmas and their parameters (e.g., oxygen, nitrogen) on the surface and bulk properties of PP films. Pressure and density of charge carriers may be varied in the broad range in the cold plasma discharges [68,69].
(vii) In-situ ageing studies of mechanical properties of plasma-treated films.
(viii) Modeling of stress–structure coupling.

## 5. Conclusions

We conclude that cold radiofrequency air plasma treatment, commonly regarded as a surface-only modification technique, induces substantial and measurable changes in the bulk mechanical behavior of polypropylene (PP) films. Both surface and bulk properties of extruded PP films are modified by cold air plasma treatment. In our experiments, PP films were exposed to radiofrequency (RF) air plasma. The plasma power was 11 W, plasma frequency was about 13 MHz, and the average pressure in the plasma chamber was approximately 1 Torr (133 Pa). Essential changes in the Young modulus, tangent modulus, maximal elongation, ultimate tensile strength (UTS), toughness, apparent contact angle, and adhesion energy of the samples were observed in our experiments. The post-plasma-treatment evolution of the aforementioned bulk and surface properties of PP films was investigated. The UTS increased as a result of cold plasma treatment from ${UTS}_{0}=(59\pm 3)\;MPa$ to ${UTS}_{PT}=(68\pm 2)\;MPa$. The toughness of the films was markedly increased as a result of the plasma treatment, from $U_{T0}=(3323\pm 400)\;MPa$ to $U_{T\_PT}=(4434\pm 400)\;MPa$. This increase is due to the growth of both the maximal elongation and UTS of the plasma-treated samples. These results are important for numerous applications of PP films, including their industrial applications in composite laminates [70]. It is noteworthy that, in contrast, X-rays, e-beam, and gamma irradiation did not change the bulk properties of PP films [71]. Plasma treatment modifies only the nanometer-thick external layer of PP films, increasing the surface energy of the films [25,26,27,28].

Cold plasma treatment of polypropylene produces significant surface oxidation and functionalization, evidenced by a ~2.7-fold increase in oxygen content and the emergence of diverse C–O, C=O, and COOH functionalities. Nitrogen incorporation (up to 1.2 at%) indicates that atmospheric species participate in the modification process. The partial compositional recovery observed between 6 and 48 h suggests dynamic surface reorganization, likely involving molecular mobility and selective migration of functional groups. It is generally agreed that these modifications substantially alter the surface chemistry while preserving the bulk polymer properties.

In contrast, we report that the cold plasma treatment of the extruded polymer films results in dramatic changes in the bulk properties of PP films. Thus, we observed an inverse Rehbinder effect, i.e., surface modification improved the bulk properties of the PP films, such as toughness and UTS. Usually, the surface modification leads to a reduction in the hardness and ductility of crystalline materials by a surfactant film. In our experiments, surface treatment of PP films resulted in the improvement of their bulk mechanical properties. That is why we designate the observed phenomenon the inverse Rehbinder effect. We relate the improvement of mechanical properties of PP to the morphological transformations in the plasma-treated PP films [50]. The bulk and surface properties of the plasma-treated PP films evolve with time. The following hierarchy of the temporal scales related to the studied relaxation processes is established: $\tau_{HR}>\tau_{\varepsilon }=\tau_{T}=\tau_{UTS}>\tau_{E}$, where $\tau_{HR}$, $\tau_{\varepsilon }$, $\tau_{T}$, $\tau_{UTS}$, and $\tau_{E}$ are the time scales of change in apparent contact angle (hydrophobic recovery), elongation, toughness, ultimate tensile strength, and Young modulus respectively. The longest of the relaxation times is related to the surface processes, i.e., hydrophobic recovery. The time scales of hydrophobic recovery and changes in the bulk properties of plasma-treated PP are well-separated. It is plausible to conclude that the bulk and surface kinetic processes occurring in the plasma-treated PP films are driven by different physical mechanisms.

Thus, we finally conclude that cold plasma processing can be used not only for adhesion promotion but also for mechanical performance tuning. Enhanced toughness and UTS are directly relevant to flexible packaging, composite reinforcement films, membranes, and biomedical substrates. The process is low-temperature, solvent-free, and scalable, making it attractive for industrial implementation. In our future research we plan to investigate the chemical modification and morphological changes in the plasma-treated PP films, including extended XRD study of crystallinity of the plasma-treated polymer films.

## Figures and Tables

**Table 1:** Comparison of the characteristic time scales, established for different parameters of the plasma-treated PP films.

Relaxation Process	Characteristic Time Scale, Hours
Relaxation of Young modulus, $\tau_{E}$	6.7±2.4
Relaxation of tangent modulus, $\tau_{tm}$	20±2.0
Relaxation of ultimate tensile strength, $\tau_{UTS}$	25.0±2.5
Relaxation of toughness, $\tau_{T}$	25.0±2.5
Relaxation of maximal elongation, $\tau_{\varepsilon }$	25.0±2.5
Relaxation of the apparent contact angle, $\tau_{HR}$	155±41

**Figure A1:**
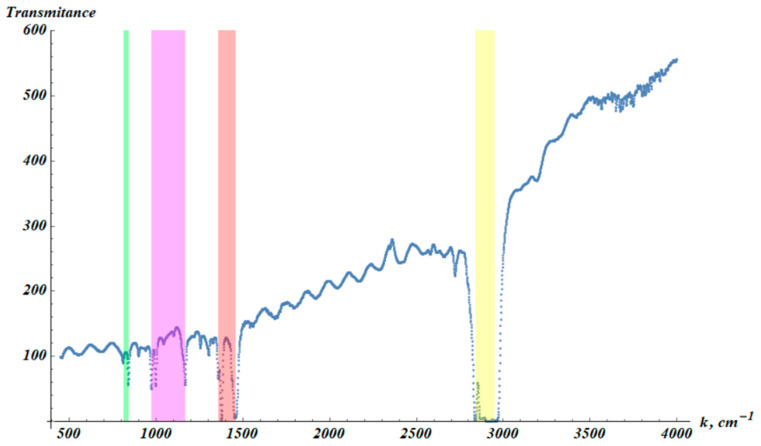
The polypropylene transmittance spectrum. The ordinate axis is the transmittance of the sample presented in arbitrary units.

## Data Availability

The original contributions presented in this study are included in the article. Further inquiries can be directed to the corresponding author.

## Abbreviations

The following abbreviations are used in this manuscript:

FTIR	Fourier-Transform Infrared spectroscopy
RF	Radio frequency
PP	Polypropylene
UTS	Ultimate tensile strength

## Appendix A

The transmittance spectrum of PP was measured with the Mid-IR FTIR spectrometer JASCO FT/IR-4600 at ambient conditions. The distinctive PP fingerprint lines can be seen in Figure A1, depicting the transmittance spectrum of PP films.

- Yellow zone: stretching vibration of CH_2_ can be observed around 2930 cm^−1^ and around 2850 cm^−1^. These lines correspond to asymmetric and symmetric stretching vibration of CH_2_. Antisymmetric and symmetric stretching vibration of CH_3_ can be recognized around 2960 cm^−1^ and 2870 cm^−1^, respectively.
- Red zone: symmetric in-plane vibration of C–H (–CH_3_) at 1455 cm^−1^ and the prominent peak around 1380 cm^−1^, which relates to CH_3_ asymmetric bending vibration seen in PP.
- Magenta zone: C–C deformation lines around 997–1000 cm^−1^ and vibrations of CH_3_ and CH_2_ groups at 970 cm^−1^.
- Green zone: C–C and C–H deformation lines around 810 cm^−1^ and 840 cm^−1^, respectively.

The transmittance FTIR spectrum of the plasma-treated PP was identical to that of virgin PP.
